# Increased Sulfiredoxin Expression in Gastric Cancer Cells May Be a Molecular Target of the Anticancer Component Diallyl Trisulfide

**DOI:** 10.1155/2019/4636804

**Published:** 2019-02-04

**Authors:** Juan Wang, Ligang Si, Genyu Wang, Zhigang Bai, Wenmei Li

**Affiliations:** ^1^Department of Respiratory Medicine, Peking University Third Hospital, Beijing 100191, China; ^2^Department of Pediatrics, The First Affiliated Hospital of Harbin Medical University, Harbin, Heilongjiang 150001, China; ^3^Institute of Nuclear and New Energy Technology, Tsinghua University, Beijing 100084, China; ^4^Department of General Surgery, Beijing Friendship Hospital, Capital Medical University, Beijing 100050, China; ^5^Laboratory of Molecular Oncology, Key Laboratory of Carcinogenesis and Translational Research (Ministry of Education), Peking University Cancer Hospital/Institute, Beijing 100142, China

## Abstract

Sulfiredoxin (Srx) is a newly discovered antioxidant enzyme playing a role in the catalytic reduction of oxidative modifications. Srx is overexpressed in a variety of cancers. It may promote carcinogenesis as well as tumor progression. In this study, we report for the first time that Srx expression might be positively associated with the development of gastric cancer and tumor malignancy. Immunohistochemistry showed that, compared to normal tissues (42%, 20/47), Srx expression in gastric tumors (85%, 40/47) was much more common (chi-square test,* p*<0.01). In addition, the staining of Srx was stronger in poorly differentiated gastric cancer than in well-differentiated gastric cancer. Western blotting showed that, in the gastric tumor cell line BGC823, the Srx protein was upregulated in response to H_2_O_2_ treatment, although it was inadequate to counteract the increased oxidative stress, as indicated by the gradually increasing level of malondialdehyde (MDA). In addition, Srx expression, MDA levels, and ROS levels in BGC823 cells were markedly inhibited upon treatment with diallyl trisulfide (DATS), a major constituent of garlic oil with proven anticancer effects. These results suggest that Srx may be an oxidative stress marker. Antioxidation may account for the anticancer potential of garlic.

## 1. Introduction

Gastric cancer is one of the most widespread cancer types and represents an important case of mortality in China [1, 2]. The treatment of gastric cancer requires a multidisciplinary approach and is usually based on surgery, radiotherapy and chemotherapy [3]. Nevertheless, even the use of the best possible approach is associated with treatment failure and mortality [4, 5]. A novel promising anticancer approach is the modulation of oxidative stress and the modulation of the antioxidant capacity of tumors [6–8]. Indeed, tumor cells are highly metabolically active and generate a high amount of reactive oxygen species (ROS) as byproducts of mitochondrial activity [6–8]. This intrinsic oxidative stress can be harmful to cancer cells in the absence of the proper defense mechanisms [6–8].

Indeed, to counteract the harmful effects of ROS and maintain oxidation-reduction (redox) homeostasis, aerobic species evolved an antioxidant system. The peroxiredoxin (Prx) family is a group of enzymes that efficiently reduce H_2_O_2_ and alkyl hydroperoxides. Srx is an enzyme that is responsible for the reversal of hyperoxidized sulfinic Prx in yeast [9, 10], mammals [10], and plants [11]. Owing to the effect of Srx, Prxs are reactivated. Thus, Srx plays a role in maintaining redox balance. Srx is critical for redox balance and the survival of cells exposed to low, steady state levels of H_2_O_2_ [12]. The Srx/Prx axis has been shown to promote lung cancer maintenance and metastasis, suggesting that it could be targeted for cancer prevention and treatment [13]. Another study revealed that Srx is probably an oncoprotein in cervical cancer and that it plays an important role in the activation the Wnt/*β*-catenin pathway, which is involved in cancer cell survival [14]. Similar results have been obtained in various cancer types [15]. The inhibition of Srx has been suggested as a strategy against cancer [16]. Nevertheless, so far, there is no study of the role of Srx in gastric cancers.

Allium vegetables have been used for hundreds of years and in multiple cultures as a multipurpose medicine [17]. Allium species are well-known for their benefits for the cardiovascular system, immune functions, blood glucose levels, radioprotection, microbial protection, and anticancer properties [17]. Diallyl trisulfide (DATS) is a compound extracted from garlic oil and has been shown to be epidemiologically responsible for the anticancer effect of garlic [18–21]. In our previous study, the human gastric tumor cell line BGC823 was treated with DATS extract from garlic and the results showed that the transcription of SH18, an analog to human Srx (*hSrx*), was inhibited by DATS. Therefore, it suggests that the inhibition of Srx-mediated antioxidant responses may be involved in the mechanisms underlying the anticancer effects of DATS.

Therefore, the present study aimed to preliminarily explore the role of Srx in gastric cancers and to study whether DATS treatment could modulate the protein levels of Srx. The results could provide some clues about novel targets against gastric cancers.

## 2. Material and Methods

### 2.1. Tissue Microarray and Immunohistochemistry

Tissue microarray and immunohistochemistry staining were constructed as previously described [22]. A total of 94 human gastric specimens including 47 gastric carcinomas and 47 nonneoplastic normal samples were obtained from the tumor bank of Beijing Cancer Hospital. Clinical tissue specimens were collected after receiving informed consent of the patients and approval of the local research ethical committee. All methods were performed in accordance with the relevant guidelines and regulations. Sections were cut from the original histopathological blocks, deparaffinized in xylene and rehydrated with a descending ethanol series. The sections were immersed in EDTA (pH 8.0) for 12 min and boiled in a microwave oven for 2 min. After blocking in 5% skimmed milk for 1 h at room temperature, the sections were incubated with anti-hSrx antibody (1:200) (self-preparation; see the section “Antibody preparation”) overnight at 4°C. After that, the sections were incubated with a goat anti-rabbit secondary antibody (1:1000) (Santa Cruz Biotechnology, Santa Cruz, CA, USA) at room temperature for 2 h, and the immunoreactivity was determined using the DAB system (Santa Cruz Biotechnology, Santa Cruz, CA, USA). The sections were counterstained with hematoxylin to indicate the nuclei.

### 2.2. Cell Culture and Grouping

The gastric tumor cell line BGC823 stored in our lab was grown in DMEM supplemented with 10% fetal bovine serum (GIBCO, Invitrogen Inc., Carlsbad, CA, USA), 100 U/ml penicillin, and 100 U/ml streptomycin at 37°C in a humidified atmosphere of 95% air and 5% CO_2_ (V/V). The BGC823 cells were grown in DMEM to a density of 1×10^6^ cells, and then 100 *μ*M H_2_O_2_ (Sigma, St Louis, MO, USA) was added to the medium. The cells were collected at 0, 0.5, and 1 h and then subjected to malondialdehyde (MDA) measurement and western blotting.

For DATS treatment, BGC823 cells were stimulated with 5 *μ*g/ml DATS (Hefeng Co. Ltd., Shanghai, China). The cells were collected at 0, 2, and 4 h and then subjected to western blotting and immunofluorescence (only for 0 and 2h).

### 2.3. MDA Measurement

Malondialdehyde (MDA) levels were measured using the thiobarbituric-acid-reactive substances (TBARS) method, according to the manufacturer's protocol (Jiancheng Biological Engineering, Nanjing, Jiangsu, China). Briefly, BGC823 cells (1×10^7^) were washed three times with PBS after incubation with DATS or H_2_O_2_, respectively. Cell lysates were obtained by three cycles of freeze-thaw at -20°C and 37°C in lysis buffer. The extract was sonicated for 30 s and centrifuged at 10,000 rpm for 10 min. The supernatant (0.05 *μ*L) was added with 200 *μ*L of 8.1% sodium dodecylsulfate and 3.0 ml of 0.4% 2-thiobarbituric acid in 10% acetic acid solution (pH 3.5). The mixture was incubated at 95°C for 60 min. After cooling, 5.0 ml of* n*-butanol and pyridine (15:1) and 1.0 ml of distilled water were added, and the mixture was centrifuged at 2000 ×g for 10 min. TBARS were measured with a spectrophotometer (Shanghai Optical Instruments, Shanghai, China) at 515 nm (excitation) and 553 nm (emission) in the butanol-pyridine phase. MDA concentration was calculated according to the product manual.

### 2.4. Immunofluorescence

After DATS treatment, BGC823 cells were cultured overnight on cover slides in 6-well plates. The cells were washed with phosphate-buffered saline (PBS; pH 7.4) and fixed with cooled acetone at 4°C for 30 min. After permeabilization with 0.5% Triton X-100 in PBS (PBST), the cells were incubated with the self-prepared anti-hSrx antibody (1:25) for 1 h at 37°C. The cells were washed extensively with 0.5% PBST before incubation with an FITC-conjugated secondary antibody (1:100; Santa Cruz Biotechnology, Santa Cruz, CA, USA) for 1 h at room temperature. The cells were washed and incubated with DAPI (1:2000) for 3 min. The cells were washed again and visualized under a confocal laser-scanning microscope.

### 2.5. Western Blotting

After treatment with H_2_O_2_ or DATS, total proteins were extracted from BGC823 cells through incubating in SDS sample buffer (50 mmol/L Tris·Cl, 2% SDS, 10% glycerol, 100 mmol/L DTT, and 0.1% bromophenol blue) at 95°C for 5 min. Protein concentration was measured using the Bradford method. About 50 *μ*g of protein was loaded onto 12% SDS-PAGE. After electrophoretic separation, the proteins were transferred onto PVDF membranes, and the membranes were incubated with self-prepared anti-hSrx antibody (1:200) or polyclonal primary antibody against actin (1:1000) (Santa Cruz Biotechnology, Santa Cruz, CA, USA). Then, the membranes were incubated with HRP-conjugated goat anti-rabbit secondary antibodies (1:1000) (Santa Cruz Biotechnology, Santa Cruz, CA, USA) at room temperature for 2 h, and signals were detected using an ECL system (GE Healthcare, Waukesha, WI, USA).

### 2.6. Detection of Reactive Oxygen Species (ROS)

ROS were detected according to the method described by Gomes [23]. Briefly, we used 2,7-dichlorodihydrofluorescein diacetate (H_2_DCFDA; Molecular Probes, Eugene, OR, USA), a specific molecular probe to measure H_2_O_2_. H_2_DCFDA diffuses through the cell membrane and is enzymatically hydrolyzed by an intracellular esterase to nonfluorescent dichlorofluorescein, which reacts with H_2_O_2_ to form a fluorescent compound. After treatment with DATS, 10 *μ*M H_2_DCFDA was added to BGC823 cells (1×10^7^) for 30 min for the detection of H_2_O_2_. Then, the cells were examined under a confocal laser-scanning microscope.

### 2.7. Antibody Preparation

A cDNA library was established through reverse transcription (Invitrogen Inc., Carlsbad, CA, USA) using total RNA from human fetal liver tissue. The complete coding sequence (CDS) of* hSrx* was cloned from the library with the forward primer 5′-GCG GAT CCA TGG GGC TGC GTG CAG GAG G-3′ and reverse primer 5′-GGG AAT TCC TAC TGC AAG TCT GGT GTG GAT-3′, containing recognition sites for the restriction enzymes* BamH*I and* EcoR*I. The amplified fragment was digested and ligated to the pGEX-KG expression vector. The recombined plasmid was transformed into* E. coli* XA90 cells for expressing the hSrx protein.

The recombinant fusion protein made of hSrx and glutathione S-transferase (GST) was purified through affinity chromatography using glutathione sepharose. The hSrx protein was confirmed by mass spectrometry. The purified protein was injected into rabbits four times to generate polyclonal antibodies against hSrx. Antibody purification was performed with antigen-immunoaffinity chromatography, in which the hSrx protein was associated with CNBr-activated sepharose 4B. Purified antibody was eluted according to the manufacturer's protocol (Amresco, Solon, OH, USA).

### 2.8. Statistical Analysis

All analyses were performed using SPSS 10 (SPSS Inc., Chicago, IL, USA). The association between Srx expression and tumor incidence was determined using the chi-square test. Two-sided P-values <0.05 were considered statistically significant.

## 3. Results

### 3.1. Srx Expression Was Increased in Human Gastric Tumors Compared with Normal Tissues

**Figure 1:**
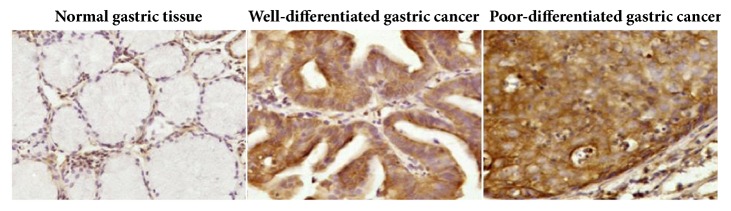
Sulfiredoxin (Srx) protein expression in gastric cancer tissues and normal gastric tissues. Clinical tissue specimens were collected from surgical resection for gastric adenocarcinoma. Immunohistochemistry was performed using a home-made antibody. The sections were counterstained with hematoxylin to indicate the nuclei. The association between Srx expression and tumor incidence was determined using the chi-square test.

We first analyzed Srx protein expression in human gastric tumors and matched normal tissues by immunohistochemistry (Figure 1). Srx was barely detectable in normal gastric tissues, but high expression of Srx protein was found in gastric tumors (Table 1). Srx was present in 85% of gastric tumors (40/47), while only in 42% (20/47) of matched normal tissues (*p*<0.01). The staining of Srx was stronger in poorly differentiated gastric cancer than in well-differentiated gastric cancer, suggesting that Srx expression may be positively associated with the malignancy of the cancer. However, expression of Srx between two types of gastric cancer did not reach significant difference (Table S1).

### 3.2. Srx Expression Was Induced upon H_2_O_2_ Treatment in the Gastric Tumor Cell Line BGC823

**Figure 2:**
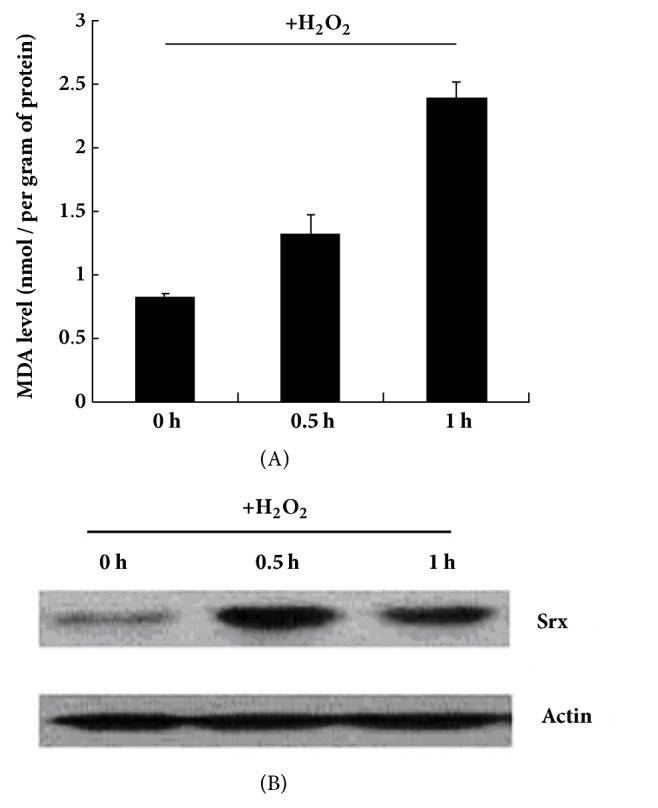
Malondialdehyde (MDA) level and Srx protein expression in BGC823 cells upon H_2_O_2_ treatment. The BGC823 cells were grown in DMEM to a density of 1×10^6^ cells, and then 100 *μ*M H_2_O_2_ was added to the medium. The cells were collected at 0, 0.5, and 1 h and then subjected to MDA measurement (A) and western blotting (B).

Upon H_2_O_2_ treatment, MDA levels gradually increased in the BGC823 cells from 0 to 1 h (Figure 2(A)), indicative of a response to oxidative stress. Srx expression was increased at 0.5 h and decreased at 1 h but was still higher than that at 0 h (Figure 2(B)).

### 3.3. DATS Treatment Decreased Srx Expression in Gastric Tumor Cell Line BGC823

**Figure 3:**
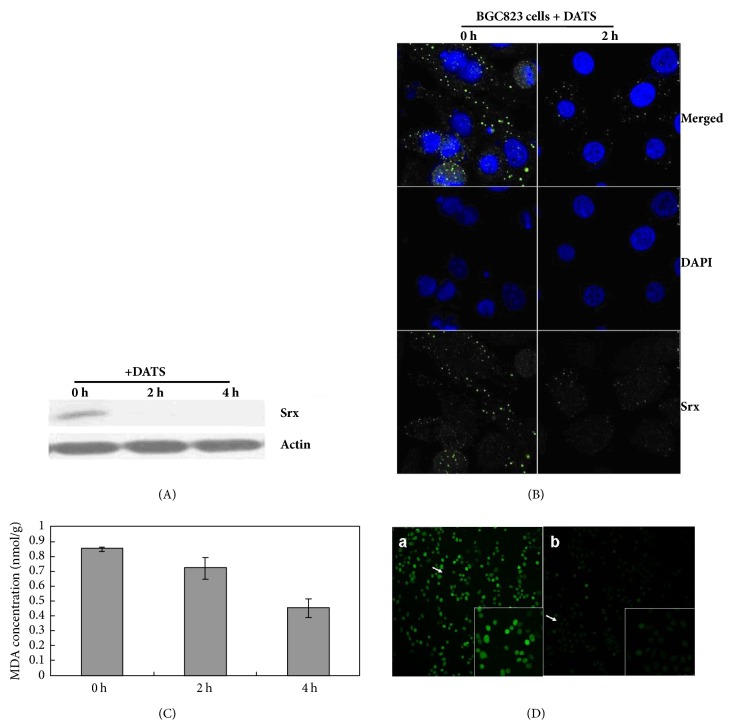
Effect of diallyl trisulfide (DATS) on Srx protein expression, MDA level and H_2_O_2_ level in BGC823 cells. (A) Western blot of Srx after 0, 2, and 4 h of 5 *μ*g/ml DATS treatment of BGC823 cells. (B) Immunofluorescence analysis of Srx showing the downregulation of Srx after 2 h of 5 *μ*g/ml DATS treatment. (C) MDA level after 0, 2, and 4 h of 5 *μ*g/ml DATS treatment of BGC823 cells. (D) H_2_DCFDA in BGC823 cells. a, control; b, 5 *μ*g/ml DATS incubation for 2 h.

Our previous study, using cDNA representative differential analysis (RDA), showed that DATS treatment could decrease Srx mRNA expression in BGC823 cells (Figure S1). Here, the present study examined the change of Srx protein expression upon DATS treatment in BGC823 cells. Western blotting showed a rapid decrease in Srx protein after 2 h of DATS treatment, and this reduction was sustained at undetectable levels after 4 h under the experimental conditions (Figure 3(A)). Similar results were obtained by immunofluorescence (Figure 3(B)). There was a significant decrease in the fluorescence intensity of Srx staining in BGC823 cells after 2 h of DATS treatment compared with 0 h. Srx was located in the cytoplasm (Figure 3(B)), which is consistent with earlier reports [9, 10]. These results corroborated our previous RDA study and further confirmed that DATS could rapidly suppress Srx at both the transcriptional and translational levels.

To further demonstrate the relationship between Srx expression and oxidation stress status, MDA, a product of lipid peroxidation, was monitored using a TBARS assay. MDA levels were decreased in BGC823 cells after treatment with 5 *μ*g/ml DATS compared with controls (Figure 3(C)). MDA levels were decreased by 15% and 50% after DATS treatment for 2 and 4 h, respectively, which may be the result of a general decrease in ROS levels.

Next, H_2_DCFDA, a specific molecular probe for H_2_O_2_, was used to detect the ROS levels in BGC823 cells. In the DATS-treated cells, the fluorescent signal induced by ROS generation in response to H_2_O_2_ was weak, whereas the signal was stronger in nontreated cells (Figure 3(D)). These results indicate that DATS treatment decreased ROS, MDA, and Srx protein levels in BGC823 cells.

## 4. Discussion

In our previous work, the cDNA RDA technique was used to identify differentially expressed mRNA [24], and SH18 was one of the downregulated mRNA in BGC823 cells following DATS treatment, as confirmed by Northern blot (Figure S1). SH18 is analogous to Srx. In this study, we found that Srx amount was higher in gastric cancer tissues than normal tissues. The protein expression of Srx was increased by oxidative stress in gastric tumor cell line BGC823. Srx expression was decreased to undetectable levels (by western blot) when the cells were treated with DATS. The variation of Srx expression was consistent with the variation in MDA levels and ROS level, in response to both DATS and H_2_O_2_ treatment.

Garlic and its extracts have been reported to enhance the antioxidant system [25] and increases the endogenous GSH levels [26]. Allicin activates the NF-E2-related factor 2 (Nrf2), which controls the defenses against oxidative stress and inflammation [27]. Garlic extract plays an anti-inflammatory role through inhibiting ROS and NF-*κ*B [28]. MDA is produced when the lipid components of cell membranes are submitted to an oxidative insult and MDA is used as an oxidative stress marker [29]. DATS treatment decreased Srx, MDA, and H_2_O_2_ levels in cells. H_2_O_2_ induces Srx expression and Srx can recover activate Prxs by reducing inactive Prx-SO_2_ [30], which then degrade H_2_O_2_. Therefore, DATS does not decrease H_2_O_2_ via decreasing Srx expression. In contrast, DATS must increase other major antioxidant [31] to decrease the H_2_O_2_ levels [32]. As a result, the low levels of H_2_O_2_ cannot induce Srx expression, which ultimately decreases Srx levels. Therefore, higher Srx levels do not mean that the antioxidative ability of cells was increased. On the contrary, Srx overexpression indicates that the ROS levels in cells surpassed the antioxidant ability, resulting in a bias toward oxidative stress conditions. Compared with the high abundance of Prx I and Prx II (nearly 0.8-1% of the total soluble proteins), the background expression of Srx was in the range of 5-10 ng/mg of the total protein lysate [6], challenging the role of Srx as a major antioxidant. Although some background expression of Srx is absolutely necessary in cells [33], the upregulation of Srx often implies an abnormal redox status. Taken together, our data indicate that Srx acts more like an oxidation stress marker than an antioxidant. Although the specific role of Srx in the process of gastric carcinogenesis remains unknown, the increase in Srx levels observed with more advanced cancer may suggest increasing oxidative stress status from normal gastric tissues to poorly differentiated gastric cancer tissues. Accordingly, Srx protein was also overexpressed in human lung tumor tissues [13, 34]. MDA is commonly used as an oxidative stress marker for detecting ROS levels and evaluating oxidative stress in vivo [29]. Srx expression correlates with the levels of MDA [35].

Allium vegetables have attracted attention as potential chemopreventive vegetables [17]. Garlic has been shown to have antitumor, antiarthritic, antidiabetic, antimicrobial, and antineurodegenerative properties [17]. Solid epidemiological evidence showed an association between the consumption of high amounts of garlic and a reduced risk of cancer [36–38], particularly gastric cancer and colorectal cancer [39, 40]. Epidemiologic studies revealed that the risk of several types of cancer [41–44] is inversely correlated with garlic intake. In the present study, DATS treatment decreased Srx, as shows by western blot, immunofluorescence and Northern blot. As expected, H_2_O_2_, MDA, and Srx are always consistent. H_2_O_2_ lead to MDA formation and induced Srx expression. Elevated Srx levels were observed in gastric tumor tissues. DATS can inhibit the formation of H_2_O_2_, MDA, and Srx. Thus, the benefits of long-term garlic intake are to enhance ROS scavenging and to maintain redox homeostasis, which ultimately prevents carcinogenesis.

Of course, the present study is exploratory and additional experiments are needed to confirm that Srx inhibition by DATS leads to decrease cancer cell initiation. In addition, further studies are necessary to determine the precise mechanisms being involved, but the Wnt/*β*-catenin pathway is probably involved [14], as well as c-Jun, AP-1 [45], Nrf2 [34], and PI3k/Akt [46]. In addition, genetic polymorphisms could be involved in the modulation of Srx levels, such as Nrf2 polymorphisms [47].

## 5. Conclusions

Oxidative stress leads to molecular damage and the accumulation of such oxidative damage ultimately results in carcinogenesis. Garlic and its extract could suppress ROS, possibly accounting for its anticancer activity.

## Figures and Tables

**Table 1:** Immunohistochemistry of Srx in gastric tumor and normal gastric tissue.

	Positive	Negative	P value
Normal	20/47 (42%)	27/47 (58%)	<0.01
Tumor	40/47 (85%)	7/47 (15%)	

## Data Availability

The data used to support the findings of this study are included within the article.

## Supplementary Materials

Supplementary Materials Table  S1: Correlation between Srx expression and clinical pathological features in gastric cancer. A total of 47 human gastric carcinomas (15 females and 32 males) were obtained, 14 of them < 60 years and the others ≥ 60 years. No correlation was observed between Srx expression level and clinical characteristics, such as age, sex, differentiation, lymph node invasion and TNM staging. Figure  S1: Northern blot of SH18. BGC823 cells were treated with DATS (5 *μ*g/ml). Total RNAs extracted from paternal BGC823 cells and BGC823 cells after exposure to DATS for 24, 48, 72, and 96 h (10 mg for each sample) were fractionated on a formaldehyde agarose gel and transferred onto nitrocellulose filters, then cross linked using an UV Stratalinker. Northern blot was performed with isotope (a-^32^P-dCTP) labeled probes of SH18 as described [24].

## References

[1] Anderson W. F., Camargo M. C., Fraumeni J. F., Correa P., Rosenberg P. S., Rabkin C. S. (2010). Age-specific trends in incidence of noncardia gastric cancer in US adults. *Journal of the American Medical Association*.

[2] Zhu X., Li J. (2010). Gastric carcinoma in China: current status and future perspectives (review). *Oncology Letters*.

[3] NCCN Clinical Practice Guidelines in oncology (NCCN Guidelines) (2017). *Gastric Cancer. Version 1.2017*.

[4] Marrelli D., Morgagni P., de Manzoni G. (2012). Prognostic value of the 7th AJCC/UICC TNM classification of noncardia gastric cancer: analysis of a large series from specialized western centers. *Annals of Surgery*.

[5] Chua T. C., Merrett N. D. (2012). Clinicopathologic factors associated with HER2-positive gastric cancer and its impact on survival outcomes-A systematic review. *International Journal of Cancer*.

[6] Trachootham D., Alexandre J., Huang P. (2009). Targeting cancer cells by ROS-mediated mechanisms: a radical therapeutic approach?. *Nature Reviews Drug Discovery*.

[7] Noh J., Kwon B., Han E. (2015). Amplification of oxidative stress by a dual stimuli-responsive hybrid drug enhances cancer cell death. *Nature Communications*.

[8] Sznarkowska A., Kostecka A., Meller K., Bielawski K. P. (2017). Inhibition of cancer antioxidant defense by natural compounds. *Oncotarget*.

[9] Biteau B., Labarre J., Toledano M. B. (2003). ATP-dependent reduction of cysteine-sulphinic acid by S. cerevisiae sulphiredoxin. *Nature*.

[10] Chang T., Jeong W., Hyun A. W., Sun M. L., Park S., Sue G. R. (2004). Characterization of mammalian sulfiredoxin and its reactivation of hyperoxidized peroxiredoxin through reduction of cysteine sulfinic acid in the active site to cysteine. *The Journal of Biological Chemistry*.

[11] Liu X. P., Liu X. Y., Zhang J. (2006). Molecular and functional characterization of sulfiredoxin homologs from higher plants. *Cell Research*.

[12] Baek J. Y., Han S. H., Sung S. H. (2012). Sulfiredoxin protein is critical for redox balance and survival of cells exposed to low steady-state levels of H_2_O_2_. *The Journal of Biological Chemistry*.

[13] Wei Q., Jiang H., Xiao Z. (2011). Sulfiredoxin-Peroxiredoxin IV axis promotes human lung cancer progression through modulation of specific phosphokinase signaling. *Proceedings of the National Acadamy of Sciences of the United States of America*.

[14] Lan K., Zhao Y., Fan Y., Ma B., Yang S., Liu Q. (2017). Sulfiredoxin May Promote Cervical Cancer Metastasis via Wnt/beta-Catenin Signaling Pathway. *International Journal of Molecular Sciences*.

[15] Mishra M., Jiang H., Wu L., Chawsheen H. A., Wei Q. (2015). The sulfiredoxin-peroxiredoxin (Srx-Prx) axis in cell signal transduction and cancer development. *Cancer Letters*.

[16] Kim H., Lee G. R., Kim J., Baek J. Y., Jo Y. J., Hong S. E. (2016). Sulfiredoxin Inhibitor Induces Preferential Death of Cancer Cells through Reactive Oxygen Species-Mediated Mitochondrial Damage. *Free Radical Biology & Medicine*.

[17] Powolny A. A., Singh S. V. (2008). Multitargeted prevention and therapy of cancer by diallyl trisulfide and related Allium vegetable-derived organosulfur compounds. *Cancer Letters*.

[18] Seki T., Hosono T., Hosono-Fukao T. (2008). Anticancer effects of diallyl trisulfide derived from garlic. *Asia Pacific Journal of Clinical Nutrition*.

[19] Zhou Y., Zhuang W., Hu W., Liu G. J., Wu T. X., Wu X. T. (2011). Consumption of large amounts of *Allium* vegetables reduces risk for gastric cancer in a meta-analysis. *Gastroenterology*.

[20] You W. C., Chang Y. S., Yang Z. T., Zhang L., Xu G. W., Blot W. J. (1991). Etiological research on gastric cancer and its precursor lesions in Shandong. *China. IARC scientific publications*.

[21] You W. C., Blot W. J., Chang Y. S., Ershow A., Yang Z. T., An Q. (1989). Allium vegetables and reduced risk of stomach cancer. *Journal of the National Cancer Institute*.

[22] Tang Z., Zhao M., Ji J. (2004). Overexpression of gastrin and c-met protein involved in human gastric carcinomas and intestinal metaplasia. *Oncology Reports*.

[23] Gomes A., Fernandes E., Lima J. L. F. C. (2005). Fluorescence probes used for detection of reactive oxygen species. *Journal of Biochemical and Biophysical Methods*.

[24] Li Y., Lu Y.-Y. (2002). Isolation of diallyl trisulfide inducible differentially expressed genes in human gastric cancer cells by modified cDNA representational difference analysis. *DNA and Cell Biology*.

[25] Sultana M. R., Bagul P. K., Katare P. B., Anwar Mohammed S., Padiya R., Banerjee S. K. (2016). Garlic activates SIRT-3 to prevent cardiac oxidative stress and mitochondrial dysfunction in diabetes. *Life Sciences*.

[26] Liu Y., Li A., Feng X., Sun X., Zhu X., Zhao Z. (2018). Pharmacological investigation of the anti-inflammation and anti-oxidation activities of diallyl disulfide in a rat emphysema model induced by cigarette smoke extract. *Nutrients*.

[27] Zhang H., Wang P., Xue Y., Liu L., Li Z., Liu Y. (2018). Allicin Ameliorates Cognitive Impairment in APP/PS1 Mice via Suppressing Oxidative Stress by Blocking JNK Signaling Pathways. *Tissue & Cell*.

[28] Feng C., Luo Y., Nian Y. (2017). Diallyl Disulfide Suppresses the Inflammation and Apoptosis Resistance Induced by DCA Through ROS and the NF-*κ*B Signaling Pathway in Human Barrett’s Epithelial Cells. *Inflammation*.

[29] Wen J. J., Yachelini P. C., Sembaj A., Manzur R. E., Garg N. J. (2006). Increased Oxidative Stress Is Correlated with Mitochondrial Dysfunction in Chagasic Patients. *Free Radical Biology & Medicine*.

[30] Rhee S. G., Woo H. A., Kil I. S., Bae S. H. (2012). Peroxiredoxin Functions as a Peroxidase and a Regulator and Sensor of Local Peroxides. *The Journal of Biological Chemistry*.

[31] Izigov N., Farzam N., Savion N. (2011). S-allylmercapto-N-acetylcysteine up-regulates cellular glutathione and protects vascular endothelial cells from oxidative stress. *Free Radical Biology & Medicine*.

[32] Javed H., Khan M. M., Khan A. (2011). S-allyl cysteine attenuates oxidative stress associated cognitive impairment and neurodegeneration in mouse model of streptozotocin-induced experimental dementia of Alzheimer's type. *Brain Research*.

[33] Wei Q., Jiang H., Baker A. (2013). Loss of sulfiredoxin renders mice resistant to azoxymethane/dextran sulfate sodium-induced colon carcinogenesis. *Carcinogenesis*.

[34] Kim Y. S., Lee H. L., Lee K. B. (2011). Nuclear factor E2-related factor 2 dependent overexpression of sulfiredoxin and peroxiredoxin III in human lung cancer. *Korean Journal of Internal Medicine*.

[35] Höhn A., Weber D., Jung T. (2017). Happily (n)ever after: Aging in the context of oxidative stress, proteostasis loss and cellular senescence. *Redox Biology*.

[36] Lau B. H. S., Tadi P. P., Tosk J. M. (1990). Allium sativum (Garlic) and cancer prevention. *Nutrition Research*.

[37] Han J. (1993). Highlights of the Cancer Chemoprevention Studies in China. *Preventive Medicine*.

[38] Iimuro M., Shibata H., Kawamori T. (2002). Suppressive effects of garlic extract on Helicobacter pylori-induced gastritis in Mongolian gerbils. *Cancer Letters*.

[39] You W.-C., Zhang L., Pan K.-F. (2001). Helicobacter pylori prevalence and CagA status among children in two counties of China with high and low risks of gastric cancer. *Annals of Epidemiology*.

[40] Gail M. H., You W.-C., Chang Y.-S. (1998). Factorial trial of three interventions to reduce the progression of precancerous gastric lesions in Shandong, China: Design issues and initial data. *Controlled Clinical Trials*.

[41] Viry E., Anwar A., Kirsch G., Jacob C., Diederich M., Bagrel D. (2011). Antiproliferative effect of natural tetrasulfides in human breast cancer cells is mediated through the inhibition of the cell division cycle 25 phosphatases. *International Journal of Oncology*.

[42] Liu Z., Li M., Chen K., Yang J., Chen R, Wang T. (2012). S-allylcysteine induces cell cycle arrest and apoptosis in androgen-independent human prostate cancer cells. *Molecular Medicine Reports*.

[43] Ng K. T., Guo D. Y., Cheng Q. (2012). A Garlic Derivative, S-allylcysteine (SAC), Suppresses Proliferation and Metastasis of Hepatocellular Carcinoma. *PLoS ONE*.

[44] Wang Y.-B., Qin J., Zheng X.-Y., Bai Y., Yang K., Xie L.-P. (2010). Diallyl trisulfide induces Bcl-2 and caspase-3-dependent apoptosis via downregulation of Akt phosphorylation in human T24 bladder cancer cells. *Phytomedicine*.

[45] Wei Q., Jiang H., Matthews C. P., Colburn N. H. (2008). Sulfiredoxin is an AP-1 target gene that is required for transformation and shows elevated expression in human skin malignancies. *Proceedings of the National Acadamy of Sciences of the United States of America*.

[46] Wang H., Sun N., Li X., Li K., Tian J., Li J. (2016). Diallyl trisulfide induces osteosarcoma cell apoptosis through reactive oxygen species-mediated downregulation of the PI3K/Akt pathway. *Oncology Reports*.

[47] Hartikainen J. M., Tengström M., Kosma V.-M., Kinnula V. L., Mannermaa A., Soini Y. (2012). Genetic polymorphisms and protein expression of NRF2 and sulfiredoxin predict survival outcomes in breast cancer. *Cancer Research*.

