# Supplementary material for: Increased Sulfiredoxin Expression in Gastric Cancer Cells May Be a Molecular Target of the Anticancer Component Diallyl Trisulfide
Source: Biomed Res Int. 2019 Feb 4;2019:4636804. doi: 10.1155/2019/4636804 (PMC6378787; doi:10.1155/2019/4636804)
Supplement: Supplementary Materials — Table S1: Correlation between Srx expression and clinical pathological features in gastric cancer. A total of 47 human gastric carcinomas (15 females and 32 males) were obtained, 14 of them < 60 years and the others ≥ 60 years. No correlation was observed between Srx expression level and clinical characteristics, such as age, sex, differentiation, lymph node invasion and TNM staging. Figure S1: Northern blot of SH18. BGC823 cells were treated with DATS (5 μg/ml). Total RNAs extracted from paternal BGC823 cells and BGC823 cells after exposure to DATS for 24, 48, 72, and 96 h (10 mg for each sample) were fractionated on a formaldehyde agarose gel and transferred onto nitrocellulose filters, then cross linked using an UV Stratalinker. Northern blot was performed with isotope (a-32P-dCTP) labeled probes of SH18 as described [24]. [file 4636804.f1.pdf]

Supplementary Material

Table S1. Correlation between Srx expression and clinical pathological features in gastric cancer.

Characteristics	n	Srx		*p* value
		positive	negative	
Age	47			
19-59 years	14	13	1	0.657
60- 87 years	33	27	6	
Sex				
Female	15	13	2	1
Male	32	27	5	
Differentiation				
Poor	37	32	5	1
Well	10	8	2	
Lymph node invasion				
Negative	17	15	2	1
Positive	30	25	5	
TNM staging				
I	8	6	2	0.303
II	11	10	1	
III	19	15	4	
IV	7	7	0	

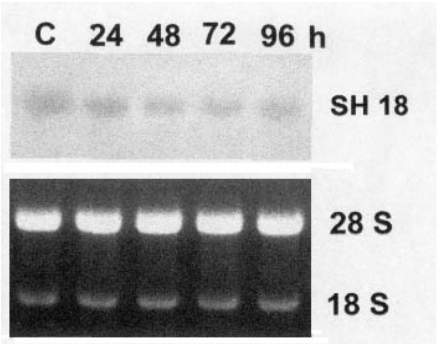

Figure S1. Northern blot of SH18.
